# Uncovering the Influence of Psychosocial Factors on Pain-Related Brain Responses in Older Adults With Chronic Pain

**DOI:** 10.1093/geroni/igaa057.2842

**Published:** 2020-12-16

**Authors:** Ellen Terry, Staja Booker, Keesha Roach, Sharon Cobb, Sheria Robinson-Lane

**Affiliations:** 1 University of Florida, Gainesville, Florida, United States; 2 The University of Florida, Gainesville, Florida, United States; 3 Charles R. Drew University of Medicine & Science, Los Angeles, California, United States; 4 University of Michigan, Ann Arbor, Michigan, United States

## Abstract

Psychosocial factors such as experiences of discrimination, pain catastrophizing and perceived stress are associated with poor osteoarthritis-related pain and disability outcomes across sex and ethnic/race groups. However, the mechanisms that mediate these psychosocial factors and knee osteoarthritis outcomes across race and sex are unclear. A cross-sectional correlational design identified the associations between everyday discrimination and clinical pain, disability and functional performance among 188 non-Hispanic Black (NHB) and non-Hispanic White (NHW) persons with knee osteoarthritis. In a serial mediated model, perceived stress and pain catastrophizing mediated the relationship between discrimination and osteoarthritis-related outcome variables in female participants. Using magnetic resonance imaging, findings suggest that experiences of discrimination differentially affect structural brain regions based on both race/ethnicity and sex in older adults with knee osteoarthritis. Given this, we are also currently investigating the extent to which pain catastrophizing on pain-related brain structure differs across race/ethnic groups in older adults with knee osteoarthritis.

